# Ash dieback epidemic in Europe: How can molecular technologies help?

**DOI:** 10.1371/journal.ppat.1006381

**Published:** 2017-07-20

**Authors:** J. Allan Downie

**Affiliations:** John Innes Centre, Norwich Research Park, Norwich, United Kingdom; THE SAINSBURY LABORATORY, UNITED KINGDOM

An epidemic of ash dieback disease has spread east to west across Europe, first being noted in Poland in 1992 [1]. The disease is caused by the ascomycete fungus *Hymenoscyphus fraxineus* (also previously known as *Chalara fraxinea* and *H*. *pseudoalbidus*). This is one of several tree pathogens and insect pests that are recent newcomers to Europe caused by worldwide movements of plants and woody materials [2]. *H*. *fraxineus* probably arrived in Eastern Europe on *Fraxinus mandshurica* (Manchurian ash) or *F*. *chinensis* (Chinese ash) trees imported to eastern Europe from the Russian Far East [3]. Although it shows few symptoms on its native hosts, *H*. *fraxineus* rapidly infects leaves of the European ash (*F*. *excelsior*), spreading to the branches and causing symptoms ranging from mild infections (Fig 1) to the death of mature trees [4]. About a quarter of *F*. *excelsior* trees in southern Sweden were found to be either dead or severely damaged and it was expected that further severe damage and tree deaths would be observed over time [5]. Here, I briefly outline the life cycle of *H*. *fraxineus*, evidence for a founder effect when it arrived in Europe, and the observation that it greatly outnumbers *H*. *albidus*, a native saprophyte on European ash. I will also outline a novel approach (associative transcriptomics) that identified genetic markers in ash linked to low disease susceptibility, which is also correlated with changes in secondary metabolites in uninfected ash leaves.

**Fig 1:**
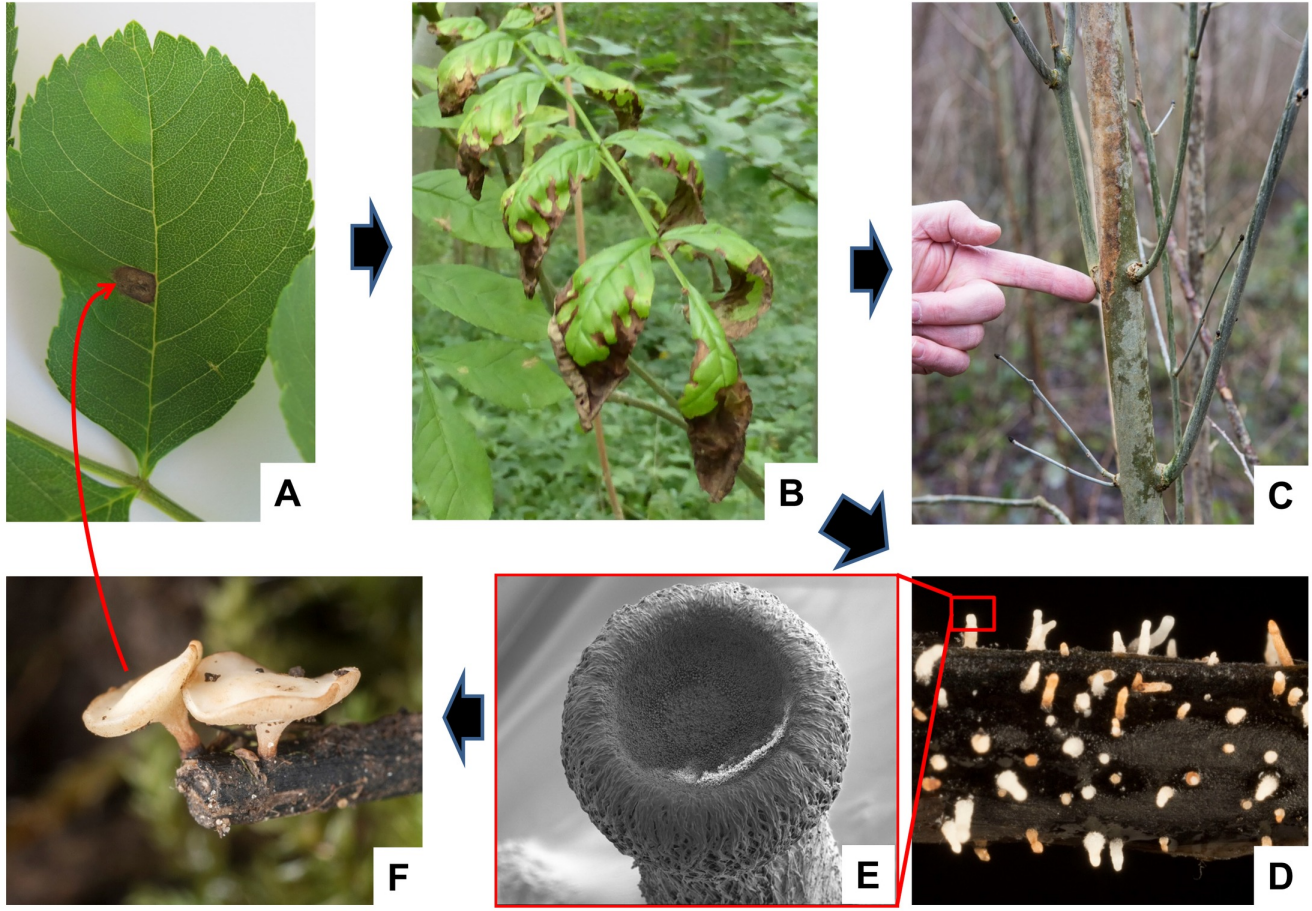
Life cycle of the *H*. *fraxineus*. **(A)** Individual ascospores of *Hymenoscyphus fraxineus* land on ash tree leaves in early summer months causing necrotic lesions. **(B)** Heavier infections cause severe symptoms on ash leaves and the fungal mycelium grows down through the leaf stems into the woody parts of the tree, where it continues to grow, causing diamond-shaped lesions **(C)** that can eventually encircle the branch, cutting off nutrient exchange with the leaves. Infected leaves are shed in autumn and by early summer, the leaf stems show early stages of growth of *H*. *fraxineus* prefruiting bodies **(D)** that are about 0.1 mm in diameter, and one is illustrated by scanning electron microscopy **(E)**. Fertilization of these, probably by conidiosore spermatia, promotes the formation of mature fruiting bodies **(F)** that fire ascospores up into the air where they are carried to ash leaves to complete the life cycle.

## A life cycle that leads to pathogen diversity

Unlike many fungal and oomycete plant pathogens that have caused major disease epidemics [6, 7], there is no evidence of clonal spread of *H*. *fraxineus*. This is due to the lifestyle of the pathogen [8, 9]. Infections are caused in the summer by (sexually produced) ascospores landing on the leaves (Fig 1), germinating, and progressively growing down the petioles into the main leaf stems (rachises). In autumn, the infected leaves and rachises are shed and the fungus overwinters among the leaf litter in the main leaf stems. In early summer, fruiting bodies (Fig 1) appear on rachises [10] and it is thought that conidiospores act as spermatia, promoting fertilisation between different strains. Mature fruiting bodies are about the size of a match head and fire ascospores into the air (typically 100,000 ascospores per m^3^ are found in infected areas) where they can be caught on the wind and so can be distributed widely [11, 12]. These sexually produced ascospores are the primary mode of dispersal, ensuring genetic diversity in the airborne spread of the disease. There is evidence that (asexual) conidiospores can also be infectious [13], possibly promoting localised spread. However, such infection must be relatively uncommon in wide dispersal of the disease, because no identical clones of infecting fungi have been identified.

The life cycle of *H*. *fraxineus* appears similar to that of the related [14] native European fungus *H*. *albidus*, which grows on but does not show pathogenic symptoms on *F*. *excelsior*. Although the fruiting bodies of *H*. *fraxineus* and *H*. *albidus* appear similar, a distinguishing feature is that, whereas *H*. *fraxineus* mating is heterothallic, *H*. *albidus* has a homothallic mating system and so shows lower genetic diversity [15]. In both species, most infections are shed with leaf fall, but *H*. *fraxineus* grows more rapidly than *H*. *albidus* in both leaf and woody material of European ash [16, 17]. This can enable some *H*. *fraxineus* infections to progress more rapidly down the leaf stem and then grow into the woody material where it can overwinter. This can cause whole branches to die in the following season [4]. Heavily damaged trees can induce the formation of new shoots from epicormic (dormant) buds that are present below the bark of branches. When this occurs on mature branches, the resulting production of new leaves can provide the fungus with direct access to mature parts of the tree via infections that can cause rapid death of the trees [18].

## A founder effect in Europe

Microsatellite markers [19], sequencing of parasitic mycoviruses [20], and genome sequencing [21] have been used to understand the population structure and genetic diversity of the invading *H*. *fraxineus* using isolates of the pathogen from Europe and Japan. The European strains are much less diverse than those from Japan and the 2 groups of isolates clustered separately, revealing that the epidemic in Europe was caused by another source of infection. The analyses point to an introduction of 2 different strains with compatible mating types, each strain being infected with a genetically distinct mitovirus, *H*. *fraxineus* MitoVirus 1 (HfMV1), that can be distinguished by RNA sequencing. Fungal mating was followed by spread of their sexual progeny throughout Europe. The high incidence (>80%) of European isolates carrying HfMV1 contrasts with the approximately 1% of Japanese isolates carrying this virus. All of this points to a founder effect in Europe and this could be due to restricted introduction due to the geographical separation of the main population of *H*. *fraxineus* in Asia or that there was a restriction that permits only a narrow range of *H*. *fraxineus* to colonise European ash. This latter explanation seems unlikely because Japanese isolates of *H*. *fraxineus* could infect *F*. *excelsior*, some appearing more virulent than the existing European isolates [16]; this raises the possibility that further Asian introductions could mate with the European population, possibly increasing pathogenicity. Another concern is that the American indigenous ash *F*. *pennsylvanica* shows some susceptibility to infection by *H*. *fraxineus*. Although it is less susceptible than *F*. *excelsior*, it indicates that this disease has the potential to spread to America [16, 17].

## Ecological displacement of the European native *H*. *albidus*?

Based on analysis of individual ascocarps and fungal isolates [22, 23], it was concluded that *H*. *fraxineus* was displacing *H*. *albidus*, which may be headed for extinction in Denmark and the Czech Republic. This is unusual and would imply that the 2 species have almost identical ecological niches. However, a weakness of such individual analyses is that if *H*. *fraxineus* greatly outnumbers *H*. *albidus* then it becomes difficult to spot *H*. *albidus*. Molecular technologies coupled with high throughput spore-trapping approaches [12, 24] allowed the characterisation of very high numbers of individuals, revealing that *H*. *albidus* was present but at orders of magnitude (10^2^ to 10^7^) less frequent than *H*. *fraxineus*. These differences in frequencies may be due in part to more rapid growth of *H*. *fraxineus* than *H*. *albidus* in leaves and woody material [16, 17], higher rates of formation of fruiting bodies, and higher rates of spore production.

## Identification of genetic markers for low susceptibility using associative transcriptomics in ash

Studies with a historical set of grafted clones of different lines of *F*. *excelsior* demonstrated that a few trees had low disease susceptibility over a wide range of environmental conditions, demonstrating a genetic basis for low susceptibility [25]. Ash is a natural out-breeder and thus mapping low susceptibility by genetic segregation would be difficult. In view of this, an approach called associative transcriptomics was used to identify genetic markers linked to low susceptibility [26]. This involves RNA sequencing a panel of diverse individuals to identify genetic markers, which are then correlated with levels of disease severity to identify those associated with inheritance of low susceptibility. This technique had been used on crop plants to identify markers linked to quality traits [27] but had not been used previously to identify markers associated with disease resistance. Two types of marker can be generated by RNA sequencing: single nucleotide polymorphisms (SNPs) and gene expression markers (GEMs) based on the abundance of different mRNA species. Using a population of ash trees with differing disease susceptibility but enriched for trees with low susceptibility, a few SNPs and GEMs were identified as being likely to be associated with trees with low susceptibility [26]. These markers were identified in young ash leaves that had not been infected and so should be correlated with some predisposition (e.g., prepriming of defence) to low susceptibility rather than simple induction of disease-resistance genes. The identified markers could be used to predict with high confidence trees that showed relatively few symptoms of ash dieback. The expression level of one of the SNP alleles was correlated with low susceptibility in European ash and was also found to be the only allele in species of *F*. *mandshurica*, *F*. *americana*, and *F*. *ornus*, all of which have low disease susceptibility [26]. A higher resolution analysis of the transcriptome data, taken in conjunction with the recently sequenced European ash genome [28], identified 20 GEMs associated with low susceptibility. Eight of these encoded MADS-box–containing proteins typical of transcription factors and 2 encoded cinnamoyl–coenzyme A (CoA) reductase 2–like genes, giving more potential genetic markers for low susceptibility. These genetic markers were identified using trees that had been exposed to the disease for up to 10–15 years and so a correlation with reduced susceptibility over a longer period remains to be established.

Using assays with 1 SNP and 3 GEMs, it appeared that the United Kingdom population of ash tested may have a higher frequency of low susceptibility than the Scandinavian ash populations tested [28]. Based on genome sequencing [28] and microsatellite markers [29], most of the UK population of *F*. *excelsior* is genetically distinct from the Scandinavian population and this could explain why rates of susceptibility may be different. Since there is genetic divergence among different subgroups of ash within Europe [28, 29], it is possible that different subgroups of *F*. *fraxineus* within Europe could respond differently to this disease.

## Disease susceptibility in ash is correlated with changes in levels of iridoid glucosides

Six of the predicted MADS-box transcripts and the 2 transcripts encoding the cinnamoyl-CoA reductase 2–like proteins were more strongly induced in ash trees of low susceptibility compared with highly susceptible trees. It was thought that a cascade of gene activation together with the different expression of the cinnamoyl-CoA reductase 2–like genes could be indicative of a change in secondary metabolites in ash. Metabolites in leaves from trees with low and high susceptibility were compared, revealing differences particularly in relation to levels of iridoid glycosides [28], which have a role in defence in the Oleaceae (to which ash belongs). However, counterintuitively, the levels of iridoid glucosides were lower in those *F*. *excelsior* trees with low susceptibility to *H*. *fraxineus*, implying it is not simply high constitutive levels of these iridoid glucosides in leaves that confer low susceptibility.

## Conclusions and future perspectives

Genome sequencing and use of DNA-based markers have given clear insights into the likely source of *H*. *fraxineus* and its high incidence of spore spread compared with *H*. *albidus* in heavily infected regions. The availability of genome sequence and various markers will enable the identification of introgression of genome regions conferring increased virulence if such developments occur. It will also be possible to measure adaptive diversity in the European population and genome comparisons (e.g., *H*. *albidus*, *H*. *fraxineus*, and other *Hymenoscyphus* spp.) may help identify key pathogenic traits. From the molecular genomics of ash trees, associative transcriptomics based on RNA sequencing is a rapid new way forward in terms of identifying genetic markers that could be used to screen for trees with low disease susceptibility. These RNA markers could be used to select saplings with increased probability of showing low susceptibility while retaining high genetic diversity, although this would be easier if DNA-based markers could be identified. Genome comparisons of lines of *F*. *excelsior* of low and high susceptibility together with genome comparison of other *Fraxinus* spp. may also lead to identification of genetic markers associated with low susceptibility. This together with analysis of changes in metabolite profiles may provide a model for dealing with future problems in trees caused by introduced diseases and pests if trees showing some degree of resistance can be identified within the native population. Ash populations are also facing another major threat due to the spread of the insect pest *Agrilus planipennis* [30], commonly known as emerald ash borer. If trees with reduced susceptibility to this insect can be identified, the molecular technologies have now been established for using associative transcriptomics and/or association genetics to identify genetic markers predicting low susceptibility.
